# Quantum Fast Forwarding of Non-Markovian Quantum Dynamics

**DOI:** 10.1021/acs.jpclett.6c02455

**Published:** 2026-10-12

**Authors:** Avin Seneviratne, Peter L. Walters, Fei Wang

**Affiliations:** † Department of Physics and Astronomy, 3298 George Mason University, 4400 University Drive, Fairfax, Virginia 22030, United States; ‡ Department of Chemistry and Biochemistry, George Mason University, 4400 University Drive, Fairfax, Virginia 22030, United States; § Quantum Science and Engineering Center, George Mason University, 4400 University Drive, Fairfax, Virginia 22030, United States

## Abstract

In this work, we present a quantum algorithm for simulating non-Markovian quantum dynamics based on the Feynman–Vernon influence functional formalism, which provides an exact description of the dynamics of a quantum system linearly coupled to a harmonic bath. In condensed-phase environments, the bath correlation functions typically decay over time, allowing them to be truncated beyond a finite time scale and enabling an iterative propagation scheme. We implement the finite-memory propagator using a fast-forwarding algorithm that achieves constant circuit depth with respect to the number of propagation steps, while the nonunitary matrices are realized on quantum circuits using unitary dilation. We further exploit the structure of the unitary dilation to show that the additional circuit complexity introduced by the dilation scales polynomially with the number of dilation ancilla qubits, while the dominant computational cost arises from the implementation of the underlying 1-dilation. The algorithm is compared with classical benchmark calculations for the spin-boson model and shows close agreement. Together, these developments establish a quantum-circuit framework for fast forwarding non-Markovian dynamics and identify the principal computational bottlenecks that must be overcome to achieve quantum advantage.

## Graphical abstract

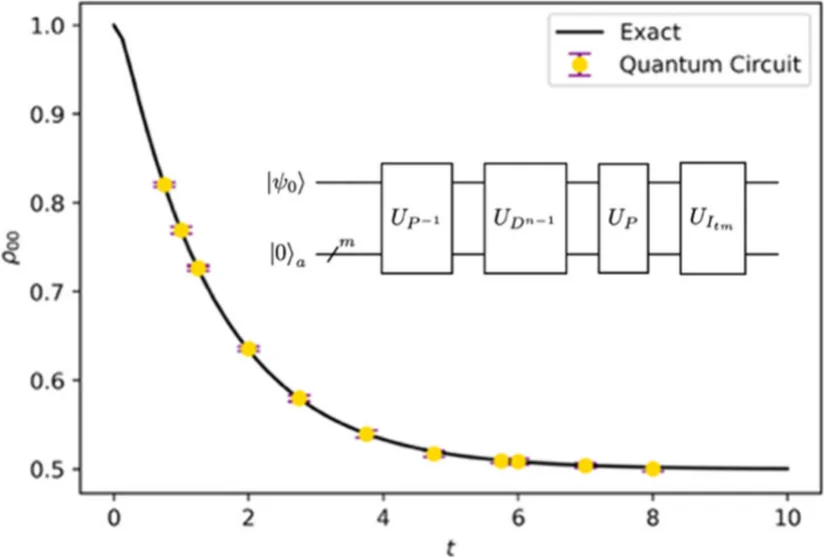

Quantum computers are rapidly advancing toward practical utility,^1,2^ with quantum simulation widely regarded as one of the most promising applications.^3–5^ The ability to efficiently represent and manipulate highly entangled quantum states makes quantum computers particularly well suited for simulating strongly correlated quantum systems.^6^ While the majority of research has focused on electronic structure problems, a growing body of work has begun to address temporal entanglement arising in quantum dynamics, specifically non-Markovian processes.^7–13^ A central challenge in developing quantum algorithms for non-Markovian quantum dynamics is how to faithfully encode the inherently nonunitary non-Markovian propagator into unitary operations executable on quantum hardware. In this work, we address this issue by developing a quantum algorithm that combines techniques of fast forwarding^14^ and unitary dilation.^15^ This construction provides a route for implementing long-time non-Markovian propagation on quantum computers with circuit depth independent of the total simulation time, for a fixed target measurement success probability. In what follows, we use the spin-boson model to demonstrate the performance of the algorithm with the dynamics obtained using the Feynman–Vernon influence functional formalism.^16^

We first introduce the theoretical framework and computational strategy. For a quantum system linearly coupled to a harmonic bath, the reduced density matrix (RDM) in Feynman’s path integral formulation is expressed as the system’s bare propagator multiplied by an influence functional:^17^

$$\rho_{s}\left(s_{N}^{+},s_{N}^{-};t\right)=\int ds_{0}^{+}\;\int ds_{1}^{+}\;\cdot \cdot \cdot \;\int ds_{N-1}^{+}\;\int ds_{0}^{-}\;\int ds_{1}^{-}\;\cdot \cdot \cdot \;\int ds_{N-1}^{-}\;<s_{N}^{+}|e^{-iH_{s}\Delta t/\hbar }|s_{N-1}^{+}>\cdot \cdot \cdot <s_{1}^{+}|e^{-iH_{s}\Delta t/\hbar }|s_{0}^{+}><s_{0}^{+}|\rho_{s}\left(0\right)|s_{0}^{-}>\times <s_{0}^{-}|e^{iH_{s}\Delta t/\hbar }|s_{1}^{-}>\cdot \cdot \cdot <s_{N-1}^{-}|e^{iH_{s}\Delta t/\hbar }|s_{N}^{-}>\times I\left(s_{N}^{+},s_{N-1}^{+},\cdot \cdot \cdot ,s_{1}^{+},s_{0}^{+},s_{N}^{-},s_{N-1}^{-},\cdot \cdot \cdot ,s_{0}^{-},s_{0}^{-};\Delta t\right)$$ 1

with the {*s*_0_^+^, *s*_1_^+^, ···, *s*_*N*_^+^} and {*s*_0_^–^, *s*_1_^–^, ···, *s*_*N*_^–^} denoting the discretized forward and backward paths, ⟨*s*_0_^+^|*ρ*_*s*_(0)|*s*_0_^–^⟩ the system’s initial condition, and Δ*t* the Trotter time step. The influence functional has the expression

2 $$I=\exp\left[-\frac{1}{\hbar }\overset{N}{\underset{k=0}{\sum }}\overset{k}{\underset{k^{\prime }=0}{\sum }}\left(s_{k}^{+}-s_{k}^{-}\right)\left(\alpha_{kk\prime }s_{k\prime }^{+}-\alpha_{kk\prime }^{*}s_{k\prime }^{-}\right)\right]$$

with the α_*kk*’_ coefficients being the discrete version of the thermally averaged bath correlation function.^18,19^ The explicit expressions for those coefficients are given in the Appendix. In continuous time, the α_*kk*’_ coefficient has the form

3 $$\alpha \left(t\right)=\frac{1}{\pi }\int_{0}^{\infty }d\omega \;J\left(\omega \right)\left[\coth\left(\frac{\hbar \omega \beta }{2}\right)\;\cos\left(\omega t\right)-i\;\sin\left(\omega t\right)\right]$$

The nonlocality of the time correlation function is responsible for the non-Markovian character of the dynamics. In condensed-phase environments characterized by broad spectral densities *J*(ω), these correlation functions generally decay over time.^18^

Implementing eq 1 on a quantum computer requires reformulating it as a matrix-vector multiplication. Here, we employ the iterative tensor scheme developed by Makri et al.,^18^ which exploits the finite memory span of the time correlation function. As a result, time propagation can be carried out by repeatedly applying the propagator to the resulting vector. We briefly summarize this scheme as follows.

Let *L*Δ*t* be the memory span of the correlation function α_*kk*’_. Define the following propagator matrix *T* that propagates *L* time steps from the initial state,

4 $$T^{\left(L\right)}\left(s_{2L-1}^{\pm },\cdot \cdot \cdot ,s_{L}^{\pm };s_{L-1}^{\pm },\cdot \cdot \cdot ,s_{0}^{\pm }\right)=\overset{L-1}{\underset{k=0}{\prod }}K\left(s_{k+1}^{\pm },s_{k}^{\pm }\right)\;I_{0}\left(s_{k}^{\pm },s_{k}^{\pm }\right)\;I_{1}\left(s_{k+1}^{\pm },s_{k}^{\pm }\right)\;I_{2}\left(s_{k+2}^{\pm },s_{k}^{\pm }\right)\cdot \cdot \cdot \;I_{L}\left(s_{k+L}^{\pm },s_{k}^{\pm }\right)$$

$I_{j}\left(s_{k+j}^{\pm },s_{k}^{\pm }\right)=\exp\left[-\frac{1}{\hbar }\left(s_{k+j}^{+}-s_{k+j}^{-}\right)\left(\alpha_{k+j,k}s_{k}^{+}-\alpha_{k+j,k}^{*}s_{k}^{-}\right)\right]$ is an element in the influence functional that dictates the coupling between two time points. Together, they account for the timewise interactions between identical, nearest, and non-nearest time points. *K*(*s*_*k*+1_^±^,*s*_*k*_^±^) = $<s_{k+1}^{+}\left|\exp\left(-\frac{iH_{s}\Delta t/\hbar }{\hbar }\right)\right|s_{k}^{+}><s_{k}^{-}\left|e^{iH_{s}\Delta t/\hbar }\right|s_{k+1}^{-}>$ is the system’s bare propagator. To match the dimensionality of the propagator matrix, the vectorized RDM is elongated (denoted by P ), with the initial condition defined as $P\left(0;s_{N-1}^{\pm },\cdot \cdot \cdot ,s_{0}^{\pm }\right)=<s_{0}^{+}\left|\rho_{s}\left(0\right)\right|s_{0}^{-}>$ . For instance, for a two-state system with memory span 2Δ*t*, the elongated density matrix with initial condition |ρ⟩ = (1,0,0,0) has the form |P>=1/2(1,0,0,0,1,0,0,0,1,0,0,0,1,0,0,0) , which can be prepared by the two Hadamard gates on the qubits representing *s*_1_^±^. The elongated RDM at time *t* = *L*Δ*t* is simply obtained through the matrix-vector multiplication,

5 $$P\left(L\Delta t;s_{2L-1}^{\pm },\cdot \cdot \cdot ,s_{L}^{\pm }\right)=\underset{s_{0}^{\pm },\cdot \cdot \cdot ,s_{L-1}^{\pm }}{\sum }T^{\left(L\right)}\left(s_{2L-1}^{\pm },\cdot \cdot \cdot ,s_{L}^{\pm };s_{L-1}^{\pm },\cdot \cdot \cdot ,s_{0}^{\pm }\right)\times P\left(0;s_{L-1}^{\pm },\cdot \cdot \cdot ,s_{0}^{\pm }\right)$$

The propagator matrix *T* ^(*L*)^ is translationally invariant in time. Therefore, once constructed for the initial time-propagation, it can be reused for subsequent iterative propagation to time *t* = (*n* – 1)*L*Δ*t* as

6 $$P\left(\left(n-1\right)L\Delta t\right)={\left\{T^{\left(L\right)}\right\}}^{n-1}P\left(0\right)$$

Unlike conventional propagation schemes that advance one time step at a time, the present matrix-vector multiplication scheme propagates the state from a one time block of duration *L*Δ*t* to the next. Finally, the vectorized RDM at *t* = *nL*Δ*t* ≡ *N*Δ*t* is obtained by

7 $$\rho_{s}\left(N\Delta t;s_{N}^{\pm }\right)=I_{0}\left(s_{N}^{\pm }\right)T^{\left(L\right)}\left(s_{N+1}^{\pm }=\cdot \cdot \cdot =s_{N+L-1}^{\pm }=0,s_{N}^{\pm };s_{N-1}^{\pm },\cdot \cdot \cdot ,s_{N-L}^{\pm }\right)P\left(\left(n-1\right)L\Delta t\right)$$

which is equivalent to

8 $$\rho_{s}\left(N\Delta t;s_{N}^{\pm }\right)=I_{tm}\cdot {\left\{T^{\left(L\right)}\right\}}^{n-1}\cdot P\left(0\right)$$

where the terminal step operators *T* ^(*L*)^ (*s*_*N*+1_^±^ = ··· = *s*_*N*+*L*–1_^±^ = 0,*s*_*N*_^±^; *s*_*N*‑1_^±^, ..., *s*_*N*‑*L*_^±^) and *I*_0_ (*s*_*N*_^±^) are combined into a single matrix *I*_*tm*_. This completes the construction of the matrix-vector multiplication scheme used to propagate non-Markovian quantum dynamics.

Next, we discuss the fast-forwarding algorithm.^14^ Rather than concatenating the *T* ^(*L*)^ circuit *m* times when doing the time evolution of eq 6, the fast-forwarding algorithm employs the decomposition of *T* ^(*L*)^ as *T* ^(*L*)^ ≈ *PDP* ^–1^, in which *D* is a diagonal matrix. As a consequence, {*T* ^(*L*)^}^*n*−1^ ≈ *PD* ^*n*^ ^–1^ *P* ^–1^, allowing the circuit depth to remain constant while only the parameters associated with *D* ^*d*^ change. When *T* ^(*L*)^ is diagonalizable, the most straightforward way to achieve such a decomposition is through eigenvalue decomposition, which we adopt in this work. Another popular approach uses quantum computers to variationally parametrize *P* and *D*, contingent on them being unitary.^14^

It should be emphasized that *T* ^(*L*)^ and *I*_*tm*_(*s*_*N*_^±^) in eq 8 are not guaranteed to be unitary, and consequently, neither are the matrices *P* and *D*. This property arises because the reduced system is subject to energy fluctuation and dissipation. Therefore, to implement these nonunitary matrices on quantum computers, we employ the theory of unitary dilation.^15,20^ For the multiplication of *r* nonunitary matrices, with *r* = 4 in the present case, each one (denoted by *Q* below) is embedded into a larger unitary matrix of *r*-degree dilation

9 $$U_{Q}=\left(\begin{array}{llllll} Q & 0 & 0 & \cdot \cdot \cdot & 0 & F_{Q^{†}}\\ F_{Q} & 0 & 0 & \cdots & 0 & -Q^{†}\\ 0 & I_{d} & 0 & \cdot \cdot \cdot & 0 & 0\\ 0 & 0 & I_{d} & \cdot \cdot \cdot & 0 & 0\\ \vdots & \vdots & \vdots & \ddots & \vdots & \vdots \\ 0 & 0 & 0 & \cdot \cdot \cdot & I_{d} & 0 \end{array}\right)$$

Here, *I*_*d*_ denotes the identity matrix block and a minimum of *r* – 1 such blocks is required. *F_Q_* = $\sqrt{I_{d}-Q^{†}Q}$ is the defect operator. If the largest singular value of matrix *Q* exceeds one, the matrix can be rescaled by its largest singular value to ensure that *F*_*Q*_ is positive semidefinite. The implementation of *U*_*Q*_ requires ⌈log_2_{(*r* + 1)*l*}⌉ number of qubits, in which *l* is the dimension of the matrix *Q*. When (*r* + 1)*l* is not a power of two, *U*_*Q*_ is padded with identity blocks to embed it into the next power-of-two-dimensional Hilbert space. Figure 1 shows the implementation of the unitary version of the product *I*_*tm*_ *PD* ^*n*^ ^–1^ *P* ^–1^.

**Figure 1:**
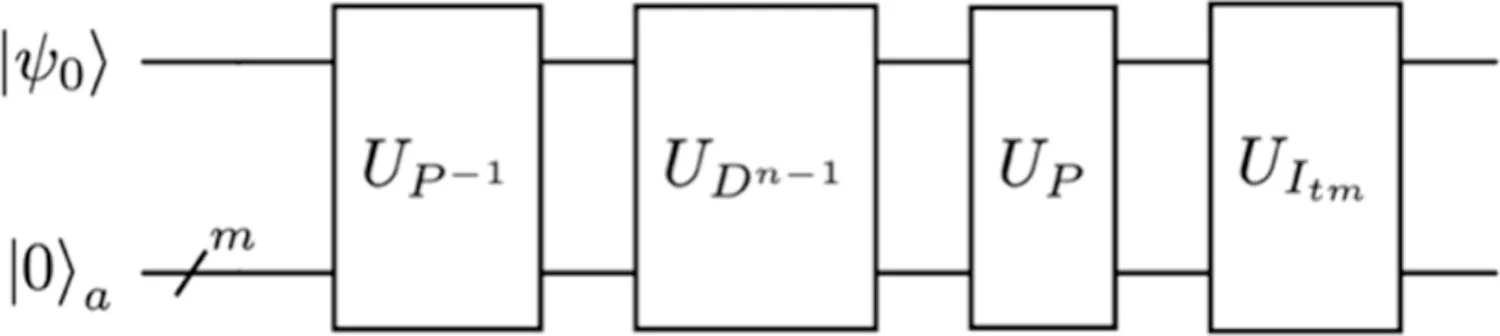
Quantum circuit for the implementation of eq 8. |ψ_0_⟩ is the initial state of the elongated RDM P(0) and |0⟩_*a*_ is the ancilla qubits required for the dilation.

The compilation of matrix *U*_*Q*_ dominates the computational cost. In what follows, we refer to the qubits which *Q* acts on as the “system qubits” and the remaining qubits as the “ancilla qubits”. For a generic unitary matrix acting on *g* number of qubits, the decomposition into elementary one- and two-qubit gates scales as *O*(4^*g*^).^21^ However, the unitary matrix of eq 9 possesses additional structure. We exploit this structure by noting that the matrix can be transformed into block-diagonal form by permuting its columns, specifically by moving the second block column to the end. Therefore,

10 $$U_{Q}=\left(\begin{array}{lllll} Q & F_{Q^{†}} & 0 & \cdots & 0\\ F_{Q} & -Q^{†} & 0 & \cdots & 0\\ 0 & 0 & I_{d} & \cdots & 0\\ \vdots & \vdots & \vdots & \ddots & \vdots \\ 0 & 0 & 0 & \cdots & I_{d} \end{array}\right)\left(\begin{array}{llllll} I_{d} & 0 & 0 & \cdots & 0 & 0\\ 0 & 0 & 0 & \cdots & 0 & I_{d}\\ 0 & I_{d} & 0 & \cdots & 0 & 0\\ 0 & 0 & I_{d} & \cdots & 0 & 0\\ \vdots & \vdots & \vdots & \ddots & \vdots & \vdots \\ 0 & 0 & 0 & \cdots & I_{d} & 0 \end{array}\right)≔U_{B}U_{perm}$$

in which *U*_*B*_ denotes the block-diagonal matrix and *U*_*perm*_ is the permutation matrix.

We first discuss the implementation of the block-diagonal matrix *U*_*B*_. It is simply the direct sum, *J*(*Q*) ⊕ *I*_*d*_ ⊕ ··· ⊕ *I*_*d*_, in which *J*(*Q*) is the 1-dilation of *Q*, consisting of blocks *Q*, *F*_*Q* ^†^_, *F*_*Q*_ and −*Q* ^†^. Denote the total number of ancilla qubits by *h* ≔ ⌈log_2_(*r* + 1)⌉, in which *r* is the degree of dilation. The 1-dilation requires one ancilla qubit, which we denote by *a*_*s*_, while the remaining ancillas are denoted by *a*_*I*_ and encode the identity blocks. This direct-sum implementation can be straightforwardly realized as an (*h* – 1)-controlled operation in which the action of *J*(*Q*) on the system and *a*_*s*_ qubits is controlled by the ancilla qubits *a*_*I*_ in the |0⟩ state. Such an (*h* – 1)-controlled operation can be decomposed into elementary gates in two ways. One approach decomposes it into 2*h* – 4 number of Toffoli gates, which requires additional *h* – 2 clean ancilla qubits, and a controlled *J*(*Q*) operation.^22^ The controlled *J*(*Q*) operation scales as *O*(4^*q*^) in the worst case, with *q* being the number of qubits required to represent the block matrix *Q*. Therefore, this implementation of *U*_*D*_ scales as *O*(4^*q*^ + *h*), with an additional *O*(*h*) ancilla overhead. Alternatively, a decomposition requiring no additional ancilla qubits can be employed. It first decomposes *J*(*Q*) into at most *O*(4^*q*^) elementary gates and then promotes each gate to an (*h* – 1)-controlled operation, with each (*h* – 1)-controlled gate scaling as *O*(*h* ^2^).^23^ This results in an overall complexity of *O*(*h* ^2^ 4^*q*^).

Next, we discuss the implementation of the permutation matrix *U*_*perm*_. It can be simplified as

11 $$U_{perm}=\left(\begin{array}{llllll} 1 & 0 & 0 & \cdot \cdot \cdot & 0 & 0\\ 0 & 0 & 0 & \cdot \cdot \cdot & 0 & 1\\ 0 & 1 & 0 & \cdot \cdot \cdot & 0 & 0\\ 0 & 0 & 1 & \cdot \cdot \cdot & 0 & 0\\ \vdots & \vdots & \vdots & \ddots & \vdots & \vdots \\ 0 & 0 & 0 & \cdot \cdot \cdot & 1 & 0 \end{array}\right)⊗I_{Q}≔\Pi ⊗I_{Q}$$

in which *I*_*Q*_ is the identity matrix with the same dimension as the block *Q*. The true permutation is encoded in the matrix Π that performs cyclic permutations on all the ancilla qubit states, while leaving the system qubit space unchanged. It essentially performs the operations through bit flips such as 000 → 001, 001 → 010, ..., 111 → 000 on ancilla states. With the first ancilla qubit taken to be the least significant bit, such a reversible cyclic increment can be implemented by a (*h* – 1)-controlled NOT gate on the last ancilla qubit and a (*h* – 2)-controlled NOT gate on the second-to-last ancilla qubit, followed by progressively lower-order controlled-NOT gates, ending with a singly controlled NOT gate on the second ancilla qubit and a NOT gate on the first ancilla qubit. The circuit therefore contains such *h* operations. Again, because a *k*-controlled NOT gate can be decomposed into *O*(*k*) Toffoli gates using clean ancilla qubits,^22^ summing the decomposition costs over control orders from zero to *h* – 1 gives an overall complexity of *O*(*h* ^2^). Alternatively, without additional ancilla qubits, each *k*-controlled NOT gate can be decomposed into *O*(*k* ^2^) elementary gates,^23^ and summing over all control orders yields an overall scaling of *O*(*h* ^3^).

Altogether, the circuit complexity for implementing the unitary matrix *U*_*Q*_ scales at most as *O*(4^*q*^) with the number of system qubits *q*, and scales either as *O*(*h* ^2^) with *h* – 2 additional ancilla qubits or as *O*(*h* ^3^) without additional ancilla qubits, where *h* is the total number of ancilla qubits required for the dilation. Equivalently, these correspond to polylogarithmic scaling with the degree of dilation *r*, as *h* = ⌈log_2_(*r* + 1)⌉. Thus, the additional circuit complexity introduced by the dilation itself scales efficiently with the number of ancilla qubits. The major cost arises from the implementation of the 1-dilation of *Q*, i.e., *J*(*Q*). The circuit for implementing *U*_*Q*_ is shown in Figure 2.

**Figure 2:**
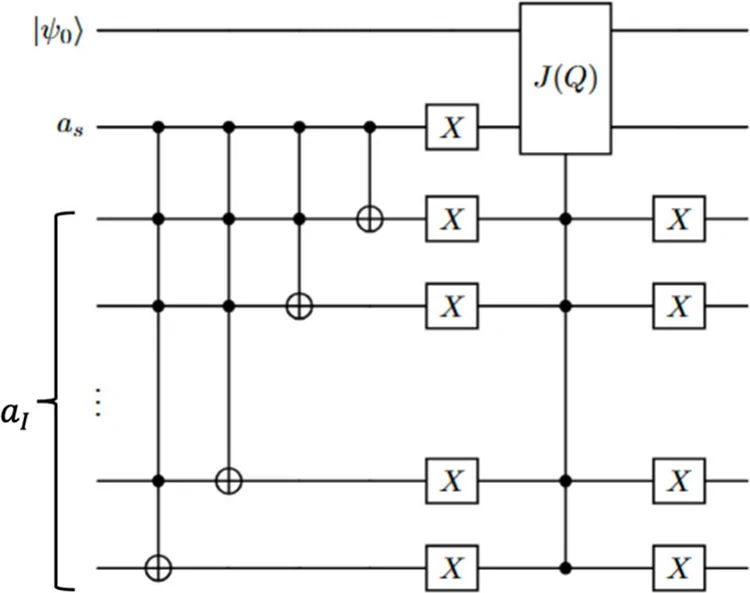
Circuit diagram for the implementation of the unitary dilation matrix *U*_*Q*_.

In what follows, we describe the measurement protocol for extracting the elements of the RDM. Since the final state vector generated by the time evolution is a superposition of all elements of the RDM, recovering each element requires projection onto a specific state. The projection can be viewed as computing the overlap between the final state vector and the basis vector of the RDM element. These overlaps can be evaluated on a quantum computer using a modified Hadamard test,^25^ shown in Figure 3. Here, *U* generates the final state produced by the quantum circuit shown in Figure 1, and *U*_*ij*_ transforms the initial computational basis into the basis vector corresponding to the RDM element. For instance, if the RDM matrix element ρ_00_ is desired, then *U*_*ij*_ (i.e., *U*_00_) is simply the identity matrix, as the computational basis (1, 0, ..., 0) encodes the ρ_00_ element. Similarly, to measure the off-diagonal elements, an *X* gate is applied to the relevant qubit. In Figure 3, only the top qubit is measured. Assigning the eigenvalues +1 and −1 to the |0⟩ and |1⟩ states, respectively, the expectation values of the measurement outcomes from Figure 3(a),(b) yield the real and imaginary parts of ⟨ψ_0_|*U*_*ij*_^†^ *U*|ψ_0_⟩ ≔ *ρ̃*_*ij*_, respectively. Here, |ψ_0_⟩ denotes the initial state of the RDM. Due to the rescaling associated with dilation and the normalization of the state vectors, *ρ̃*_*ij*_ is proportional, but not identical, to the RDM element *ρ*_*ij*_. The rescaling and normalization introduce a common multiplicative factor for all RDM elements. Therefore, for every measurement outcome *ρ̃*_*ij*_, we renormalize it to obtain the true value of *ρ*_*ij*_ according to *ρ*_*ij*_ = *ρ̃*_*ij*_/∑_*i*_ *ρ̃*_*ii*_. It should be pointed out that the multiplicative factor, regardless of its magnitude, does not affect the final result of *ρ*_*ij*_ in principle, as the common factor in the numerator and denominator cancels out. In practice, however, the accuracy depends on how faithfully the measurement reproduces the true value of *ρ̃*_*ij*_.

**Figure 3:**
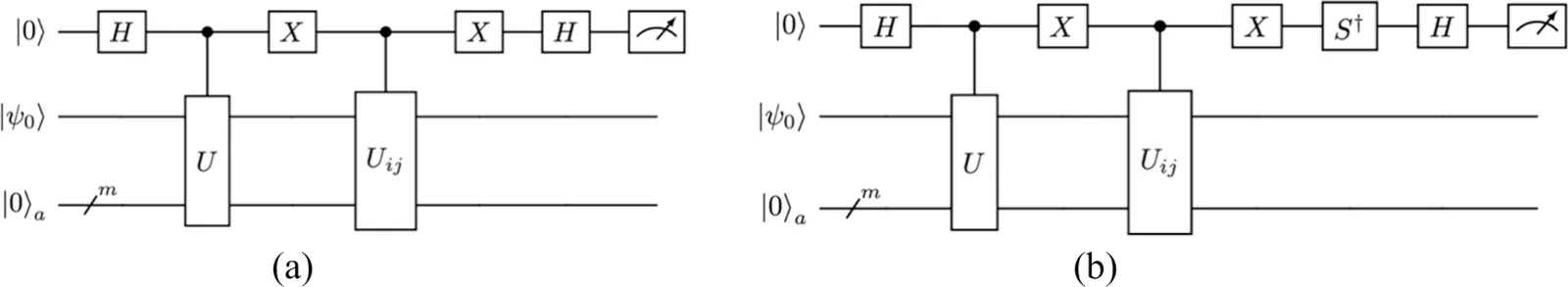
Quantum circuits for extracting the real (a) and imaginary part (b) of the RDM.

When the amplitude of the target state is small, the amplitude amplification (AA) algorithm^26^ can be used to boost the success probability. The corresponding AA circuit is shown in Figure 4. Each AA iteration consists of two reflections. In our case, these correspond to reflections about the state generated by *U*_*ij*_ (the element of the RDM) and |ψ⟩ (the final state generated by the time-evolution circuit in Figure 1). These two reflections can be expressed as

12 $$G≔S_{\psi }S_{\rho_{ij}}$$

in which

$$S_{\rho_{ij}}=2U_{ij}{|0>}^{⊗n}{<0|}^{⊗n}{U_{ij}}^{†}-I$$ 13

and

$$S_{\psi }=2U{|0>}^{⊗n}<{0|}^{⊗n}U^{-1}-I$$ 14

**Figure 4:**
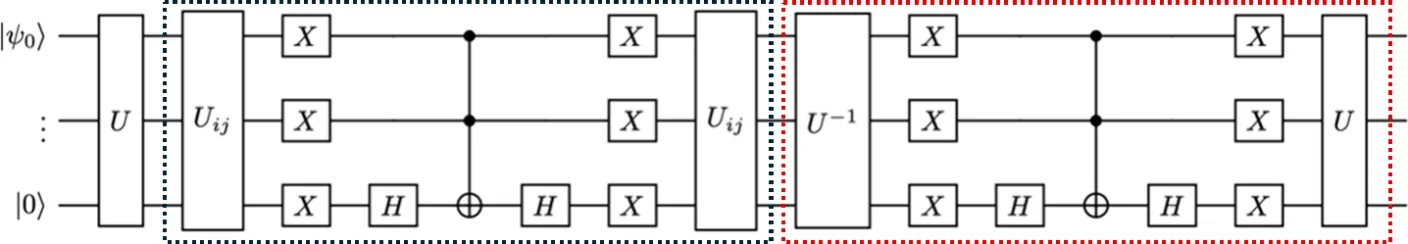
Quantum circuit for one Grover iteration. The black dashed box encloses the circuit implementing *S*_*ρ*_*ij*__, while the red dashed box encloses the circuit implementing *S*_*ψ*_.

Here, I is the identity operator and *U* is the dilated unitary that generates the final state. The AA is implemented by repeatedly applying *G*, until the desired success probability is achieved.

We now present simulation results obtained using the proposed quantum algorithm. The spin-boson model is used as the benchmark system. Specifically, the system Hamiltonian describes a symmetric two-level system with a nonzero off-diagonal coupling,

$$H_{s}=-\hbar \Omega \left(|s_{1}><s_{2}|+|s_{2}><s_{1}|\right)$$ 15

where |*s*_1_⟩ and |*s*_2_⟩ are eigenstates of the position operator *ŝ*,

$$ŝ|s_{i}>=s_{i}|s_{i}>$$ 16

and Ω is the tunneling frequency. The harmonic bath and its interaction with the system are characterized by the Ohmic spectral density,

17 $$J\left(\omega \right)=\frac{\pi }{2}\hbar \xi \omega e^{-\omega /\omega_{c}}$$

where the parameter ξ determines the strength of the system-bath coupling and *ω*_*c*_ is the cutoff frequency of the bath. The simulations are performed in two representative parameter regimes. One corresponds to weak system–bath coupling, low temperature, and fast bath dynamics, with Ω = 1, ξ = 0.1, β = 5, *ω*_*c*_ = 7.5 (Figures 5 and 6). The other corresponds to strong system–bath coupling, high temperature, and moderately fast bath dynamics, with Ω = 1, ξ = 1.2, β = 0.2, *ω*_*c*_ = 2.5 (Figures 7 and 8). The memory spans are chosen to be Δ*t* (Figures 5 and 7) and 2Δ*t* (Figures 6 and 8), respectively. The figures present the population dynamics and the real and imaginary parts of the RDM. The quantum circuits are simulated using Qiskit’s AerSimulator, and the resulting RDM elements show close agreement with the classical reference results (labeled “Exact” in Figures 5–8) obtained using the tensor-network path integral (TNPI) method.^27^ The same memory span is used in the TNPI and quantum-circuit simulations.

**Figure 5:**
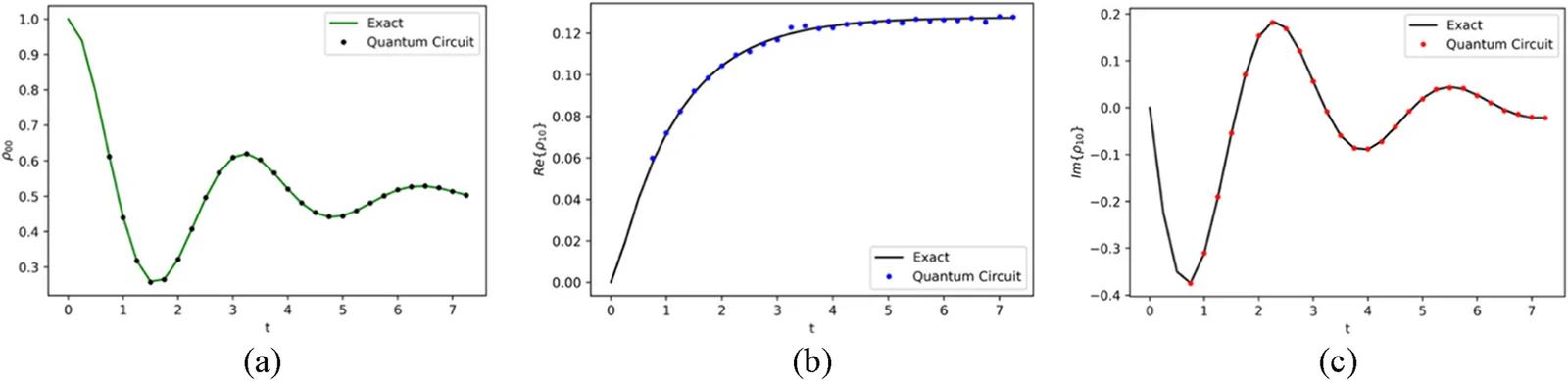
Quantum simulation of the spin-boson dynamics using Qiskit’s AerSimulator, with parameters Ω = 1, ξ = 0.1, β = 5, *ω*_*c*_ = 7.5, and Δ*t* = 0.25. The system is initially prepared in a localized state, and the memory span is Δ*t*. Each data point is obtained from 10^6^ measurement shots. (a) Population dynamics. (b) Real part of the RDM. (c) Imaginary part of the RDM.

**Figure 6:**
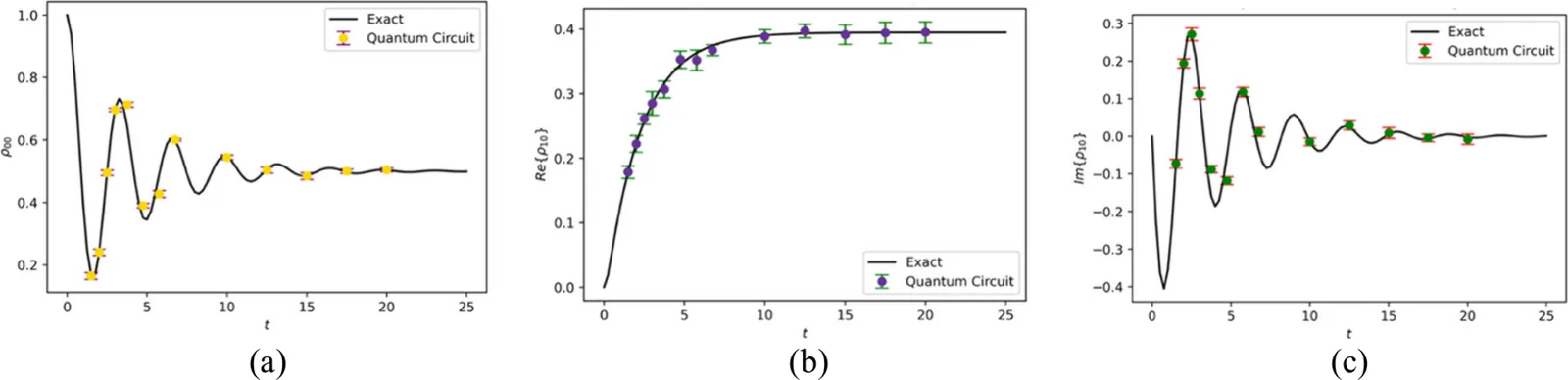
Quantum simulation of the spin-boson dynamics using Qiskit’s AerSimulator, with parameters Ω = 1, ξ = 0.1, β = 5, *ω*_*c*_ = 7.5, and Δ*t* = 0.25. The system is initially prepared in a localized state, and the memory span is 2Δ*t*. Each data point is obtained by averaging over 10 independent runs. In each run, 500 Grover iterations are performed, followed by measurement with 10^8^ shots. (a) Population dynamics. (b) Real part of the RDM. (c) Imaginary part of the RDM.

**Figure 7:**
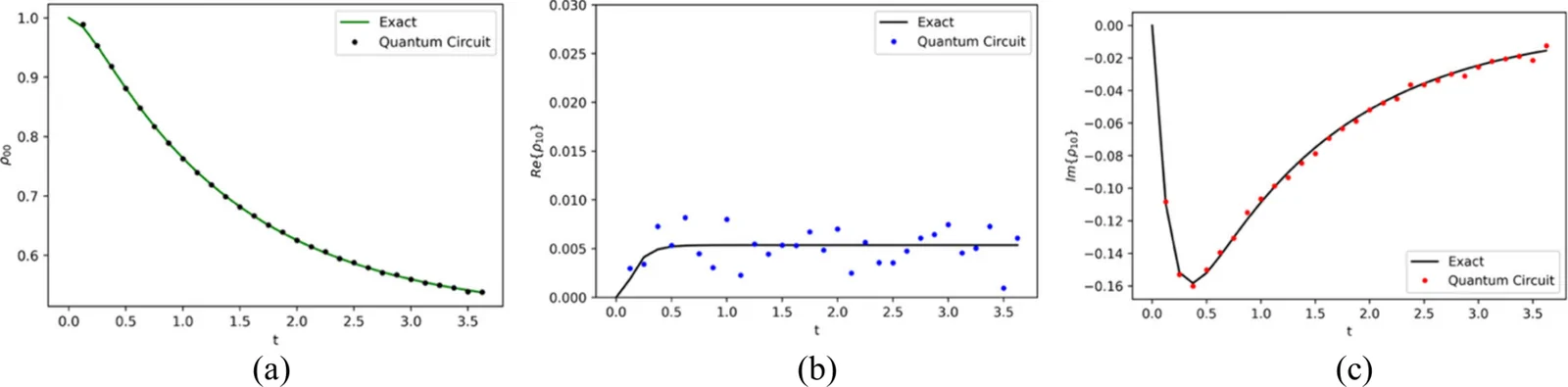
Quantum simulation of the spin-boson dynamics using Qiskit’s AerSimulator, with parameters Ω = 1, ξ = 1.2, β = 0.2, *ω*_*c*_ = 2.5, and Δ*t* = 0.125. The system is initially prepared in a localized state, and the memory span is Δ*t*. Each data point is obtained from 10^6^ measurement shots. (a) Population dynamics. (b) Real part of the RDM. (c) Imaginary part of the RDM.

**Figure 8:**
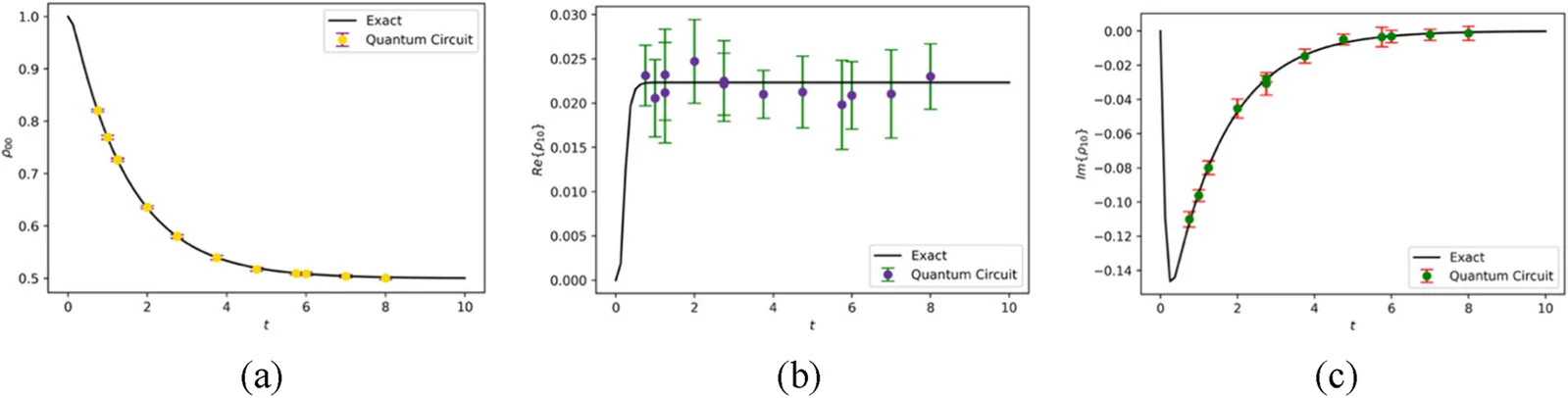
Quantum simulation of the spin-boson dynamics using Qiskit’s AerSimulator, with parameters Ω = 1, ξ = 1.2, β = 0.2, *ω*_*c*_ = 2.5, and Δ*t* = 0.125. The system is initially prepared in a localized state, and the memory span is 2Δ*t*. Each data point is obtained by averaging over 10 independent runs. In each run, 500 Grover iterations are performed, followed by measurement with 10^8^ shots. (a) Population dynamics. (b) Real part of the RDM. (c) Imaginary part of the RDM.

For the case of memory span Δ*t*, the elongated RDM has the form $P\left(s_{k}^{\pm }\right)$ , which contains four states and therefore requires two system qubits: one for the two forward states and one for the two backward states. The implementation in Figure 1 involves the multiplication of four matrices and therefore requires a 4-dilation, introducing three additional qubits. Thus, a total of five qubits is required. For the case of memory span 2Δ*t*, the elongated RDM has the form $P\left(s_{k}^{\pm },s_{k+1}^{\pm }\right)$ , which requires two additional qubits to represent the additional time step. The number of dilation qubits remains unchanged, as the dilation degree remains 4. Therefore, the total number of qubits in this case is seven. In general, for an *n*-level system with a memory span of *L*Δ*t*, there are *n* ^2*L*^ elements in the elongated RDM, requiring ⌈2*Llog*_2_ *n*⌉ number of qubits. Adding the three ancilla qubits for the dilation, the total number of qubits is ⌈2*Llog*_2_ *n*⌉ + 3, which scales linearly with the memory length and logarithmically with the system dimension.

To illustrate the practical compilation cost and the benefit of exploiting the dilation structure, Table 1 compares two strategies for implementing the unitary dilation *U*_*P*_ shown in Figure 1: direct compilation of the full unitary matrix and compilation of the structured circuit shown in Figure 2. Both implementations are compiled into the gate set {*CZ*, *id*, *R*_X_, *R*_Z_, *SX*, *X*} used by the *IBM_Miami* quantum computer. The results show that explicitly exploiting the dilation structure substantially reduces the compiled circuit depth for the examples considered here. Similar reductions are observed when the same structured construction is applied to the other unitary blocks in Figure 2.

**Table 1:** Comparison of Circuit Complexity Using Different Compilation Strategies

	Direct compilation		Figure 2 circuit compilation	
Memory span	Circuit depth	No. of Cz gates	Circuit depth	No. of Cz gates
Δ*t*	1102	438	473	183
2Δ*t*	18993	7608	8754	3130

The case of memory span 2Δ*t* requires amplitude amplification due to the large rescaling factor induced by the dilation. The rescaling factor in this case is on the order of 10^4^ arising primarily from *P* ^–1^. As a result, the amplitude is suppressed by a factor of 10,000. An additional factor that may contribute to the smallness of the amplitude is the intrinsically small value of the RDM element itself, which can be on the order of 10^–2^, as seen in Figure 8(b). Consequently, the desired state might have an amplitude as small as 10^–6^. Suppose we aim to amplify such an amplitude to the order of 10^–3^, the required number of Grover iterations is given by *n*_*rot*_ = ⌈1/2­(arcsin 10^–3^ /arcsin 10^–6^ −1)⌉, which is approximately 500. The amplitude *ρ̃*_*ij*_ prior to amplification can be traced back by *ρ̃*_*ij*_ = sin {arcsin­(*χ*_*ij*_)/(2*n*_*rot*_ + 1)}, in which *χ*_*ij*_ is the measurement outcome obtained from circuits in Figure 3 after the amplitude amplification. The reason that the measurement result from the Hadamard test can be used directly in this equation is that the Hadamard test estimates the real or imaginary part of the amplitude directly, rather than its squared magnitude. Based on the binomial statistics of the measurement outcomes, achieving a target relative uncertainty of 10% requires approximately *N*_*shot*_ = (1 – χ^2^)/(χ^2^ *ε* ^2^) number of shots, in which χ is the amplitude measured by the Hadamard test and *ε* is the relative uncertainty. Here, we set χ = 10^–3^ and *ε* = 10%, giving *N*_*shot*_ = 10^8^. We additionally note that this estimate corresponds to a worst-case scenario. For many data points, the situation can be significantly more favorable, as illustrated in Figure 6 and Figure 8(a),(c). Finally, to ensure accuracy, we perform multiple runs and take the average (Figures 6 and 8), which, according to the law of large numbers, converges toward the true value as the number of runs increases. It remains unclear whether the large rescaling factor is universal or specific to the parameters considered here, which warrants further investigation.

Compared with the two other methods developed by our group for implementing the Feynman–Vernon influence functional,^9,12^ the present approach requires a constant number of qubits ⌈2*Llog*_2_ *n*⌉, determined by the memory length *L* and system dimension *n*, independent of the total simulation time. In contrast, the number of ancilla qubits required by the other two methods grows with simulation time, introducing an additional source of diminishing measurement success, separate from the reduction caused by the rescaling factor. Therefore, the present work can be viewed as a direct improvement upon the quantum propagation scheme introduced in ref 9. On the other hand, we acknowledge the limitations of the present work associated with the implementation of the 1-dilation sub-block, which, in the worst case, scales exponentially with the number of system qubits. In addition, the singular value decomposition required to construct the defect operators introduces a classical computational overhead. However, these limitations may be mitigated by exploiting the underlying structure of the problem. Although a comprehensive solution is beyond the scope of the present work, we outline several promising approaches to address these issues. In the present implementation, the tensor propagator *T* ^(*L*)^ is treated as a generic dense matrix. However, *T* ^(*L*)^ is written as a product of low-dimensional influence-functional factors involving at most two time slices. Exploiting this factorized structure may allow *T* ^(*L*)^ to be implemented as low-dimensional matrices directly, each acting on a small subset of qubits. Alternatively, the dilated matrices may be represented by variational quantum circuits containing a polynomial number of parametrized gates, with the parameters optimized to reproduce the desired nonunitary blocks.^14,28^ Such an approach could circumvent the high cost of generic-unitary compilation if the structured tensor propagator admits a reasonable polynomial-depth representation. On a final note, a general nonunitary matrix is not guaranteed to be diagonalizable and may be defective. Even when the matrix is diagonalizable, strong non-normality can lead to highly nonorthogonal eigenvectors and an ill-conditioned eigenvector matrix *P*, increasing the sensitivity of the *PDP* ^–1^ construction to numerical perturbations and circuit synthesis errors. The variational *PDP* ^–1^ parametrization is therefore expected to be most reliable when the propagator is diagonalizable and its eigenvector basis is sufficiently well-conditioned. For the systems considered here, the condition numbers of *P* are *O*(1) for the case of memory span Δ*t* and *O*(10^5^) for 2Δ*t*. No significant loss of accuracy associated with this condition number is observed in the present simulations. Whether iterative tensors from the influence functional are generally diagonalizable and possess well-conditioned eigenvector bases remains to be determined.

In summary, we have developed a quantum algorithm that provides a quantum-circuit realization of iterative non-Markovian quantum dynamics. The algorithm builds upon the Feynman–Vernon influence functional formalism to describe the dynamics of the quantum system. By exploiting the finite temporal span of the memory kernel, the time evolution can be formulated as an iterative matrix–vector multiplication. The resulting non-Markovian propagator is decomposed into a matrix–diagonal–inverse-matrix structure and subsequently dilated into unitaries to enable its implementation on quantum computers. Consequently, the iterative propagation is reduced to updating only the diagonal matrix while leaving the circuit depth unchanged. The higher-degree dilation can be implemented efficiently, with the circuit depth scaling at most cubically with the number of ancilla qubits and polylogarithmically with the degree of dilation. All elements of the reduced density matrix are obtained through the modified Hadamard test by measuring only a single ancilla qubit. Comparison with classical reference results of the spin-boson dynamics confirms the validity of the proposed approach. Future work will explore structure-aware and variational implementations of the tensor propagator toward reducing both its quantum compilation cost and classical computational overhead.

## Appendix

The coefficients appearing in the influence functional in eq 2 are given in eqs [Disp-formula eqA.1]–[Disp-formula eqA.6].

$$\alpha_{k\prime k}=\frac{2}{\pi }\int_{-\infty }^{\infty }d\omega \frac{J\left(\omega \right)}{\omega^{2}}\frac{\exp\left(\frac{\beta \hbar \omega }{2}\right)}{\sinh\left(\frac{\beta \hbar \omega }{2}\right)}\sin^{2}\left(\frac{\omega \Delta t}{2}\right)\times e^{-i\omega \Delta t\left(k-k\prime \right)},,0<k<k^{\prime }<N$$
A.1

A.2
$$\alpha_{kk}=\frac{1}{2\pi }\int_{-\infty }^{\infty }d\omega \frac{J\left(\omega \right)}{\omega^{2}}\frac{\exp\left(\frac{\beta \hbar \omega }{2}\right)}{\sinh\left(\frac{\beta \hbar \omega }{2}\right)}\left(1-e^{-i\omega \Delta t}\right),,0<k<N$$

A.3
$$\alpha_{N0}=\frac{2}{\pi }\int_{-\infty }^{\infty }d\omega \frac{J\left(\omega \right)}{\omega^{2}}\frac{\exp\left(\frac{\beta \hbar \omega }{2}\right)}{\sinh\left(\frac{\beta \hbar \omega }{2}\right)}\sin^{2}\left(\frac{\omega \Delta t}{4}\right)\times e^{-i\omega \left(t-(\Delta t/2)\right)}$$

A.4
$$\alpha_{00}=\alpha_{NN}=\frac{1}{2\pi }\int_{-\infty }^{\infty }d\omega \frac{J\left(\omega \right)}{\omega^{2}}\frac{\exp\left(\frac{\beta \hbar \omega }{2}\right)}{\sinh\left(\frac{\beta \hbar \omega }{2}\right)}\left(1-e^{-i\omega \Delta t/2}\right)$$

A.5
$$\alpha_{k0}=\frac{2}{\pi }\int_{-\infty }^{\infty }d\omega \frac{J\left(\omega \right)}{\omega^{2}}\frac{\exp\left(\frac{\beta \hbar \omega }{2}\right)}{\sinh\left(\frac{\beta \hbar \omega }{2}\right)}\sin\left(\frac{\omega \Delta t}{4}\right)\times \sin\left(\frac{\omega \Delta t}{2}\right)e^{-i\omega \left(k\Delta t-(\Delta t/4)\right)},,0<k<N$$

A.6
$$\alpha_{Nk}=\frac{2}{\pi }\int_{-\infty }^{\infty }d\omega \frac{J\left(\omega \right)}{\omega^{2}}\frac{\exp\left(\frac{\beta \hbar \omega }{2}\right)}{\sinh\left(\frac{\beta \hbar \omega }{2}\right)}\sin\left(\frac{\omega \Delta t}{4}\right)\times \sin\left(\frac{\omega \Delta t}{2}\right)e^{-i\omega \left(t-k\Delta t-(\Delta t/4)\right)},,0<k<N$$

The spectral density is extended to the negative frequencies defined as *J*(−ω) = *J*(−ω) to avoid the singularity in the integration.
